# α-Chaconine and α-Solanine Inhibit RL95-2 Endometrium Cancer Cell Proliferation by Reducing Expression of Akt (Ser473) and ERα (Ser167)

**DOI:** 10.3390/nu10060672

**Published:** 2018-05-25

**Authors:** Ayşe Kübra Karaboğa Arslan, Mükerrem Betül Yerer

**Affiliations:** Department of Pharmacology, Faculty of Pharmacy, Erciyes University, Kayseri 38039, Turkey

**Keywords:** α-chaconine, α-solanine, *Solanum tuberosum* L., API-1, MPP dihydrochloride, RL95-2, endometrium cancer, steroidal glycoalkaloids, Akt, ERα

## Abstract

The aim of this study is to investigate the potential inhibitory effect of α-chaconine and α-solanine on RL95-2 estrogen receptor (ER) positive human endometrial cancer cell line and to identify the effect of these glycoalkaloids on the Akt signaling and ERα. The cell proliferation profiles and the cytotoxicity studies were performed by Real-Time Cell Analyzer (xCELLigence) and compared with Sulphorhodamine B (SRB) assay. The effects of α-chaconine (2.5, 5, 10 µM), α-solanine (20, 30, 50 µM), API-1 (25 µM) and MPP (20 µM) effects on Akt (Ser473) and ERα (Ser167) expressions evaluated by Western blot and qPCR method. Their IC_50_ values were as α-chaconine (4.72 µM) < MPP (20.01 µM) < α-solanine (26.27 µM) < API-1 (56.67 µM). 10 μM α-chaconine and 20, 30 and 50 μM α-solanine were effective in decreasing p-Akt(Ser473)/Akt ratio compared to positive control API-1. When the p-ERα/ERα ratios were evaluated, it was observed that α-chaconine (2.5, 5, 10 μM) and α-solanine (50 μM) were as effective as the specific ERα inhibitor MPP in reducing the ratio of p-ERα/ERα compared to the control group. In conclusion, it has been shown that the proliferation of α-chaconine and α-solanine in human endometrial carcinoma cells reduces the expression and activity of the Akt and ERα signaling pathway.

## 1. Introduction

Glycoalkaloids, a class of nitrogen-containing steroidal glycosides, are biologically active secondary plant metabolites, usually found in plants of the genus Solanum. These plants include many agricultural plants, especially the plants from Solanaceae family such as *Solanum tuberosum* L. (potato), *Solanum lycopersicum* (tomato) and *Solanum melongena* (eggplant) [1]. *S. tuberosum* L. (potato) contains significant amount of glycoalkaloids α-chaconine and α-solanine, which are trisaccharide steroidal glycoalkaloids [2].

**Figure 1:**
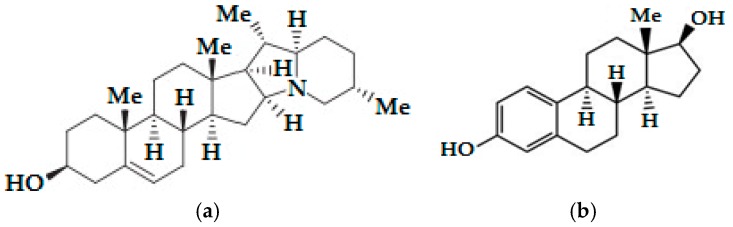
Structural similarities of the potato aglycone solanidine to the estradiol: (**a**) The molecular structure of solanidine; (**b**) The molecular structure of estradiol.

*S. tuberosum* glycoalkaloids are naturally produced and the main glycoalkaloids are α-chaconine and α-solanine, which make up 95% of the total glycoalkaloid content. The molecules of both substances contain the same steroid scaffold (aglycone), but differ in the trisaccharide moiety [1]. Steroidal glycoalkaloids are produced from the cytosolic terpenoid (mevalonate) biosynthetic pathway. Cholesterol is produced starting from acetyl-CoA. Cholesterol is modified by various glycoalkaloid metabolism enzymes via hydroxylation, oxidation and transamination, and thus solanidine (Figure 1a) is synthesized in *S. tuberosum.* Aglycone solanidine is glycosylated by solanidine glycosyltransferase enzymes to produce α-solanine and α-chaconine [3,4]. α-Chaconine and α-solanine are derived from the compound solanidine and include a carbohydrate side-chain; the side-chain is composed of glucose and two rhamnose molecules and glucose, rhamnose and galactose molecules, respectively [5]. The glycoalkaloids are found at all parts of the potato plant. The highest glycoalkaloid level is in flowers and sprouts while the lowest glycoalkaloid level is in potato tubers. Glycoalkaloids are concentrated in the skins and the prolonged exposure of the tubers to the light promotes the formation of glycoalkaloids on the surface of the tuber [1]. Steroidal alkaloids and their glycosides are known to possess a variety of biological activities including anti-tumor [6], anti-fungal [7], anti-inflammatory [8], teratogenic [9], anti-viral [10], antimicrobial [11], antiestrogenic [12,13], antiandrogenic [14] and anti-cancer activities [15]. There are some studies related to the evaluation of α-chaconine and α-solanine which are naturally occurring toxic steroidal glycoalkaloids in potato sprouts effects on different cancer cells such as lymphoma and lung [16], prostate [17], cervical [18], stomach [19], melanoma [20], pancreatic [21], breast [22] and colorectal [23].

Endometrial cancer is the fifth most common gynecological malignancy in the world in 2012, but its incidence varies between regions [24,25]. In 2012, 320,000 new cases of endometrial cancer were diagnosed worldwide. The number of new cases is expected to increase by about 70% over the next 20 years [26]. Approximately 30% of patients diagnosed with endometrial cancer are under 54 years of age, with 20% between 45–54 years of age and approximately 9% under the age of 44. For this reason, it is necessary to carefully select women with fertility-protective approaches in managing endometrial cancer [27]. Because early detection and treatment modalities have not had a major influence on mortality and fertility, searching out and developing novel approaches for treatment of endometrial cancer is very important. 

Akt, a serine-threonine protein kinase, central signaling molecule in the PI3K pathway and plays an important role in controlling the balance between cell survival and apoptosis [28]. Alterations in the Akt pathway have been identified in a variety of human cancer types, including endometrial cancer. Akt is activated by phosphorylation, on account of this Akt phosphorylation is a marker for the activation of this enzyme. The serine/threonine kinase Akt pathway integrates both extracellular and intracellular oncogenic signals, and therefore presents a promising new target for molecular therapeutics [29]. The central negative regulator of the Akt signaling cascade is the tumor suppressor gene PTEN. Mutations in PTEN result in notably increased Akt activity. PTEN mutations occur in 50% of endometrial carcinomas. Patients with increased Akt phosphorylation as a result of loss of PTEN expression have a poor prognosis. Consequently, targeting the Akt pathway may be an appropriate strategy in addressing endometrial cancer [30]. Selective inhibition of Akt represents a potential approach for the treatment of endometrium cancer. Akt/protein kinase B inhibitor, 4-amino-5,8-tetrahydro-5-oxo-8-(β-D-ribofuranosyl)pyrido[2,3-d]pyrimidine-6-carboxamide (API-1) is a novel, small molecule and potent selective inhibitor of Akt signaling that binds to the pleckstrin homology (PH) domain of Akt. It blocks its membrane translocation, which cause inhibition of Akt-regulated cell growth and cell survival *in vitro* and *in vivo* [31,32].

ERα plays a role in mediating the activity of estrogen [33]. The biological effects of 17β-estradiol (E2) (Figure 1b) are mainly mediated by ERα and estrogen receptor β (ERβ) receptors and ERα is primarily expressed in the uterus. E2 stimulates the growth of cancer cells as well as many type of cells [34]. Oncogenic transformation of cells in the uterus can initiate to the development of cancerous lesions and these lesions take advantage of E2 signaling pathways for growth [35]. Methyl-piperidino-pyrazole (MPP) is a highly selective ERα antagonist [36].

Although α-chaconine and α-solanine as being steroidal glycoalkaloid exhibit anti-carcinogenic potential against several cancer cell lines, its effect on endometrium cancer is still unclear. The aim of this study is to investigate the potential inhibitory effect of α-chaconine and α-solanine to clarify the potential of inhibiting proliferation, and to explain the effect of these glycoalkaloids on the Akt signaling and estrogen receptor α (ERα) in RL95-2, which is estrogen receptor (ER) positive human endometrial cancer cell line.

## 2. Materials and Methods

### 2.1. Materials

α-Chaconine (Cat No: A9544) was purchased from Applichem (Darmstadt, Germany), whereas α-solanine (Cat No: S3757) was Sigma (St. Louis, MO, USA). Chemical inhibitors API-1 (Cat No: 3897) and 1,3-Bis(4-hydroxyphenyl)-4-mtehyl-5-[4-(2-piperidiylethoxy) phenol]-1H-pyrazole dihydrochloride (MPP dihydrochloride) (Cat No: 1991) were purchased from Tocris bioscience (Bristol, UK). Stock solutions of α-chaconine, α-solanine and the chemical inhibitors were prepared in dimethyl sulfoxide (DMSO). DMSO (Cat No: A3672) and phosphate-buffered saline (PBS) (Cat No: A9177) were purchased from Applichem. Dulbecco’s modified eagle’s medium with F-12 nutrient mixture (DMEM: F-12) (Cat No: 01-170-1A) and penicillin/streptomycin solution (Cat No: 03-031-1C) were obtained from Biological Industries (Cromwell, CT, USA). Fetal bovine serum (FBS) (Cat No: S0115) obtained from Biochrom (Cambridge, UK). Insulin (Cat No: I9278), RNAzol (Cat No: R4533, Sigma) and trypsin-EDTA (T3924) were purchased from Sigma. Sulforhodamine B (SRB) sodium salt (Cat No: sc-253615A) and radio immuneprecipitation assay (RIPA) buffer (Cat No: sc-24948) were purchased from Santa Cruz Biotechnology (Heidelberg, Germany). Akt (Cat No: 9272), phospo-Akt (Ser473) (Cat No: 9271) and β-actin (Cat No: 4967) antibodies, estrogen receptor α (D8H8) rabbit mAb (Cat No: 8644), phospho-estrogen receptor α (Ser167) (D1A3) rabbit mAb (Cat No: 5587) and HRP-linked secondary antibody (Cat No: 7074) and BCA kit (Cat No: 7780) were purchased from Cell Signaling Technology, Inc (Leiden, The Netherlands). Reagents for electrophoresis and Western blotting were obtained from Sigma. Polyvinylidene difluoride (PVDF) membrane obtained from (Cat No: 162-0177) Bio-Rad (Dubai, United Arab Emirates). Chemiluminescence solution (ECL) (Cat No: 34080) obtained from Thermo Scientific (Paisley, UK). FastStart Essential DNA Probes Master (06 402 682 001), Transcriptor High Fidelity cDNA Synthesis Kit (05 081 955 001), RealTime ready human β-actin, Akt and ER-α catalog assays (05 532 957 001), E-plate (05 232 368 001), Cedex Smart Slides (05 650 801 001) were purchased from Roche (Roche, Laval, QC, Canada).

### 2.2. Cancer Cell Line and Culture

Human endometrial cancer cell lines RL95-2 was purchased from the American Type Cell Collection (Cat No: CRL-1671^™^ ATCC, Manassas, VA, USA). The cell line was grown in DMEM:F-12 that was supplemented with 10% (*v*/*v*) FBS, 1% penicillin/streptomycin and 0.005 mg/mL insulin. The cells were cultivated in 75 cm^2^ culture flasks at 37 °C in humidify atmosphere of 95% air and 5% CO_2_. When the cells had approximately reached 80% confluence, trypsin containing 0.25% EDTA was used to remove them from the flasks for subculture or for the corresponding experimental treatments. An inverted microscope was used to observe the changes in cell morphology. α-Chaconine, α-solanine, API-1 and MPP dihydrochloride were dissolved in DMSO and the final DMSO concentration in the medium was less than 0.1%.

### 2.3. Cell Growth and Proliferation Assay Using xCELLigence Real-Time Cell Analysis (RTCA)

The essence of the real-time growth profile is based on measuring the change in the adhesive properties of the cells as they are attached to the microelectrode-coated surface of special e-plates of xCELLigence system. Impedance measurement gives time quantitative information about the current state of cells such as cell number, viability, morphology and movement. Measurement is possible without using any label or chemicals. By eliminating the need for label or dye, it is possible to maximize the acquired data physiologically. The device’s software allows real-time monitoring of the experiment and real-time data visualization and analysis functions [37,38].

RL95-2 cells were resuspended in the culture medium and then seeded, to e-plate of xCELLigence RTCA SP system (ACEA Biosciences Inc., San Diego, CA, USA). In this experiment, different number of cells varying from 1.250 to 40.000 were used to obtain growth profile of RL95-2 cell line. xCELLigence system calculates the impedances parameter called “Cell Index (CI)”. CI values of RL95-2 were used to examine cell profile depending on proliferation and viability during 96 h. All experiments were performed at least three times.

### 2.4. Cytotoxicity Assay Using xCELLigence RTCA

According to SRB assay results, novel concentrations were determined in order to evaluate its effects on cell growth and proliferation of α-chaconine (1–10 μM), α-solanine (10–30 μM), API-1 (10–25 μM) and MPP dihydrochloride (5–50 μM). In brief, RL95-2 cells were seeded at a density of 20.000 cells per well of a 96-well E-plate (Catalog No. 05232368001; ACEA Biosciences, Inc., San Diego, CA, USA). The compounds added to cells at their growth phase. The assay was monitored during 96 h. Effects of α-chaconine, α-solanine, API-1 and MPP dihydrochloride on cell growth curve was assessed with the RTCA system xCELLigence. After obtaining cell profile data, another RTCA assay was performed by using 20.000 cells/100 μL per well to monitor the cytotoxicity of α-chaconine, α-solanine, API-1 and MPP dihydrochloride. Proliferation, spreading and cell attachment kinetics were monitored every 15 min. IC_50_ values were defined as the inhibition of the cell line by the compounds at 24 h. The RTCA software performs a curve-fitting of selected “sigmoidal dose–response equation” to the experimental data points and calculates logarithmic half maximum effect of concentration (log [IC_50_]) values at a given time point based on log of concentration producing 50% reduction of CI value relative to solvent control CI value (100%), expresses as log IC_50_ [37,38]. All experiments were performed at least three times and the results were given as the mean ± Standart Deviation (SD) of independent experiments. 

### 2.5. Determination of Cell Viability via Sulphorhodamine B Assay

The effect of α-chaconine, α-solanine, API-1 and MPP dihydrochloride on RL95-2 cell viability were determined by the method of SRB assay [39,40]. The compounds were dissolved in DMSO to a stock concentration of 50 mM. 20.000 cells/wells seeded and after 24 h incubation cells treated with different final concentrations of α-chaconine (1 nM-100 μM), α-solanine (1 nM-100 μM), API-1 (1–100 μM) and MPP dihydrochloride (1–100 μM) for 24 h, followed by fixing the cells in 10% (*v*/*v*) of trichloroacetic acid (TCA) for 1 h at 4 °C. After washing 5 times, cells were exposed to 0.5% (*w*/*v*) SRB solution for 30 min in a dark place and subsequently washed with 1% (*v*/*v*) acetic acid. After drying, 10 mM (pH 10.5) Tris base solution was used to dissolve the SRB-stained cells using a plate-shaker (PST-60 HL plus Biosan) and color intensity was measured at 510 nm in a microplate reader (Biotek Synergy HT). Data are represented as a percentage of control cells. The measurement of the “half maximal inhibitory concentration” (IC_50_) values was calculated with the Microsoft Excel program. All experiments were performed at least three times and the results were given as the mean ± SD of independent experiments.

### 2.6. Protein Extraction and Western Blot Analysis

1 × 10^6^ cells were seeded in 6 well plate to be left overnight and incubated with α-chaconine (2.5; 5; 10 μM), α-solanine (20; 30; 50 μM), API-1 (25 μM) and MPP dihydrochloride (20 μM) for 24 h. The cells were scraped using a cell scraper and centrifuged at 10.000 rpm for 10 min, after which the sample was rinsed again with PBS. Proteins were isolated with RIPA buffer. After cell lysates sonication, total protein contents were determined by using a BCA kit. A 10% separating and 5% stacking gel was prepared freshly on western blotting day. Electrophoresis was carried out at a voltage of 70 V, which was then raised to 100 V after the specimens had reached the separation gel. PVDF membrane was activated in 100% methanol for 10 s. The transfer was performed at 100 V for 1 h and 20 min in cold conditions. 5% non-fat dry milk in tris buffered saline with tween (TBST) was used for blockage for 1 h. Membranes were washed with TBST three times for 10 min. 1:1000 dilution of primary rabbit antibodies in TBST were applied to membrane and incubated overnight at 4 °C. The membranes were subsequently washed with TBST and incubated with a 1:2000 dilution of secondary antibody for 2 h at room temperature. After washing the membrane three times for 10 min in TBST, the proteins were visualized using ECL. Blue sensitive X-ray film was used for detection in a dark room. A Developer, water and fixer were used for photographic process. The band intensity was analyzed using Image J (ImageJ 1.48, ABD). β-actin was used for normalization. All experiments were performed three times and the results were given as the mean ± SD of independent experiments.

### 2.7. RNA Isolation and Quantitative Real-Time Polymerase Chain Reaction (qRT-PCR)

RL95-2 cells were seeded at a density of 1 × 10^6^ cells per well of a 6-well plate and cells were harvested for RNA isolation 24 h after the treatment with α-chaconine (2.5; 5; 10 μM), α-solanine (20; 30; 50 μM), API-1 (25 μM) and MPP dihydrochloride (20 μM). Total RNA was isolated using RNAzol in accordance with the manufacturer’s instructions. The RNA concentration and purity were determined by measurement of absorbance at 260 and 280 nm using a NanoDrop (DeNovix) and 1000 ng of each total RNA sample was used for cDNA synthesis with the Transcriptor High Fidelity cDNA Synthesis Kit (Roche, Quebec, Canada) according to the manufacturer’s specifications. qRT-PCR was performed using the FastStart Essential DNA Probes Master (Roche, Quebec, Canada) with primers. The qRT-PCR primer sequences were as follows: *ACTB 5*′-TCCTCCCTGGAGAAGAGCTA-3′ (forward) and 5′-CGTGGATGCCACAGGACT-3′ (reverse); *AKT1*, 5′-GCAGCACGTGTACGAGAAGA-3′ (forward) and 5′-GGTGTCAGTCTCCGACGTG-3′ (reverse). *ESR1*, 5′-TTACTGACCAACCTGGCAGA-3′ (forward) and 5′-ATCATGGAGGGTCAAATCCA-3′ (reverse). Threshold cycle (C_T_) values obtained from the instrument’s software were used to calculate the fold change of the respective mRNAs. ∆∆C_T_ for each mRNA was calculated by subtracting the C_T_ value of the control from the experimental value. Fold change was calculated by the formula 2^−∆∆CT^. All experiments were performed at least three times and the results were given as the mean ± SD of independent experiments.

### 2.8. Statistical Analysis

All calculations from xCELLigence were obtained using the RTCA-integrated software of the xCELLigence system. Statistical analysis was performed GraphPad Prism Software Version 7.01 (La Jolla, CA, USA) using to compare differences in values between the control and experimental group. The results are expressed as the mean ± SD. Statistically significant values were compared using one-way ANOVA and Dunnett’s post-hoc test, and *p*-values of less than 0.05 were considered statistically significant.

## 3. Results

### 3.1. Real Time Cell Growth Profile Curve

A cell number titration experiment is necessary to assess cell growth and proliferation and to decide the optimum cell number for further steps of the study. This approach not only helps to determine the appropriate seeding density but also the time for compound addition [37].

**Figure 2:**
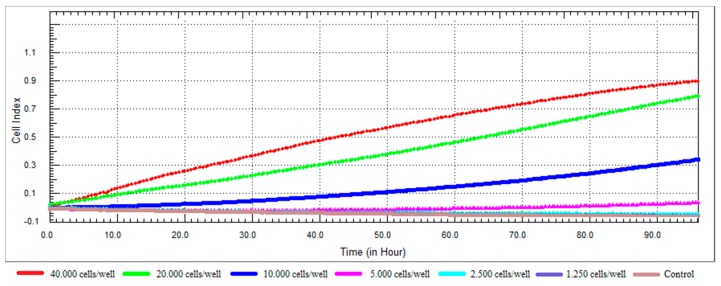
Dynamic monitoring of cell adhesion and proliferation using the xCELLigence system. RL95-2 cell at a density of 40,000; 20,000; 10,000; 5000; 2500; 1250 cells/well in E-Plates 96 were observed during 96 h.

The dynamic cell proliferation of the RL95-2 cells was monitored for a period of 96 h, and the growth curves at different densities are shown in Figure 2. When we examined the growth profile of RL95-2 cells, it was seen that the CI values were low. The factors affecting the value of CI may include cell dimension, morphology and cell-substrate adhesion quality [37,41]. Lack of CI fluctuation in cell attachment and spreading stages was characteristic for RL95-2 cells. The growth curve after cell seeding showed a linear adhesion period that was followed by a continuous increase of CI for all tested densities. According to the growth profile of the cells, even after 96 h, it has been observed that the 20,000 cells/well did not reach the plateau and continued to proliferate at a constant rate. Densities of 10,000 cells/well and above were not considered suitable for further studies as their logarithmic phase lasted before than 20,000 cells/well. It was decided that the optimal cell number for the surface area of 0.20 cm^2^/well was 20,000 cells/well. The density of 20,000 cells/well produced the most ideal growth curve for further experiments. Approximately 24-h after seeding was selected as the ideal time for compound addition.

### 3.2. Effects of the Compounds on the Cell Viability

**Figure 3:**
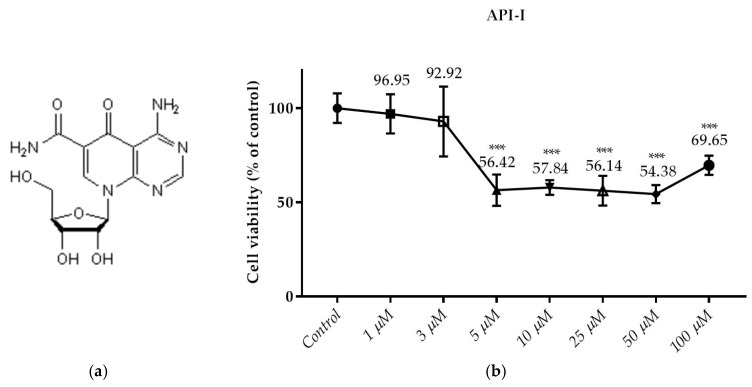
Dose dependent cytotoxic effects of API-1 was determined by SRB assay and shown for 24 h. (**a**) The molecular structure of API-1; (**b**) Effect of API-1 on viability of RL95-2 cell. Cells were treated with various concentrations of API-1 for 24 h. The absorbance was determined after 1 h-incubation of the cells with SRB. Cell viability is presented as the mean ± SD at least three independent experiments. *** *p* < 0.001 compared with the untreated control.

**Figure 4:**
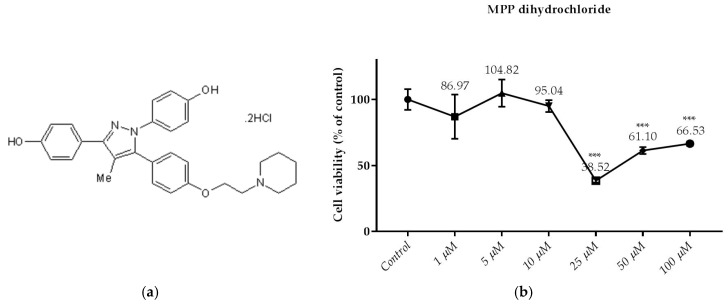
Dose dependent cytotoxic effects of MPP dihydrochloride was determined by SRB assay and shown for 24 h: (**a**) The molecular of MPP dihydrochloride; (**b**) Effect of MPP dihydrochloride on viability of RL95-2 cell. Cells were treated with various concentrations of MPP dihydrochloride for 24 h. The absorbance was determined after 1 h-incubation of the cells with SRB. Cell viability is presented as mean ± SD at least three independent experiments. *** *p* < 0.001 compared with the untreated control.

**Figure 5:**
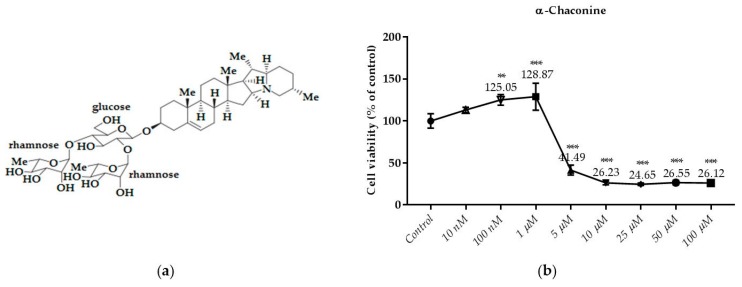
Dose dependent cytotoxic effects of α-chaconine was determined by SRB assay and shown for 24 h: (**a**) The molecular structure of α-chaconine; (**b**) Effect of α-chaconine on viability of RL95-2 cell. Cells were treated with various concentrations of α-chaconine for 24 h. Cell viability is presented as mean ± SD at least three independent experiments. ** *p* < 0.01 and *** *p* < 0.001 compared with the untreated control.

**Figure 6:**
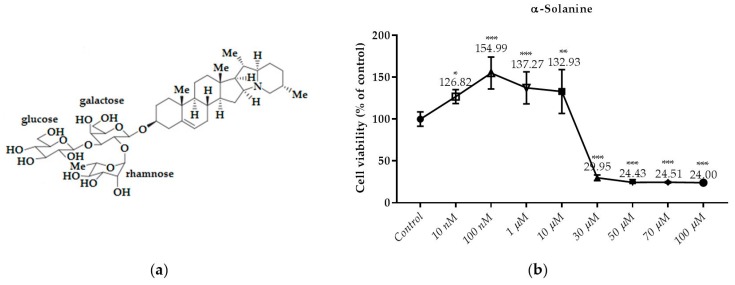
Dose dependent cytotoxic effects of α-solanine was determined by SRB assay and shown for 24 h: (**a**) The molecular structure of α-solanine; (**b**) Effect of α-solanine on viability of RL95-2 cell. Cells were treated with various concentrations of α-solanine for 24 h. Cell viability is presented as mean ± SD at least three independent experiments. ** *p* < 0.01 and *** *p* < 0.001 compared with the untreated control.

This test is the first test elucidated the cytotoxic effect of API-1, MPP dihydrochloride, α-chaconine and α-solanine on RL95-2 endometrium cancer cells (Figure 3, Figure 4, Figure 5 and Figure 6b). The cell viabilities in the presence of various concentrations of these compounds were analyzed by SRB assay. Seven different concentrations (1, 3, 5, 10, 25, 50 and 100 µM) of API-1, six different concentrations (1, 5, 10, 25, 50 and 100 µM) of MPP dihydrochloride, eight different concentrations (10 and 100 nM, 1, 5, 10, 25, 50 and 100 µM) of α-chaconine and α-solanine were selected to assess the effect of the compounds on cell viability over a wide range.

As shown in Figure 4b, the treatment of MPP dihydrochloride with 25; 50 and 100 µM for 24 h decreased cell viability significantly. However, cell viability was not significantly changed by MPP dihydrochloride at concentration below 25 µM.

At 1, 3, 5 and 10 μM concentrations there was no significant decrease compared to the control, whereas the cell viability did not change at higher than 5 µM. The next step was to work with a lower concentration of 25 μM, as it reduced viability at the same significance compared to the control at 25 and 50 μM concentration. In addition, the RTCA viability test results show a similar profile at concentrations of 10 and 25 μM, but supporting this, 25 μM concentration when evaluated together with the SRB assay results.

As shown in Figure 3b, the treatment of API-1 (5; 10; 25; 50 and 100 µM) for 24 h decreased cell viability significantly. However, cell viability was not significantly changed by API-1 at concentration below 5 µM.

As shown in Figure 5b, the treatment of α-chaconine with 5; 10; 25; 50 and 100 µM for 24 h decreased cell viability significantly. However, cell viability was not decreased by α-chaconine at concentrations below 5 µM.

α-Chaconine at concentrations of 5 μM and over were found to reduce viability in the same sense of significance compared to the control. However, at concentrations of 10 μM and higher it was considered cytotoxic since it reduced viability by more than 70% whereas α-chaconine decreased the viability by about 50% compared to the control at the concentration of 5 μM.

As shown in Figure 6b, the treatment of α-solanine with 30; 50; 70 and 100 µM for 24 h decreased cell viability significantly. However, cell viability did not decrease by α-solanine at concentration below 30 µM. It was found that the cytotoxicity produced by α-Solanine at 50 μM and higher concentrations did not increase due to the dose. A significant increase in α-solanine was observed at 10 μM compared to the control, and a decrease in viability was observed at 30 μM and over at the same concentrations.

### 3.3. Monitoring of Cytotoxicity in Real-Time Using xCELLigence System

**Figure 7:**
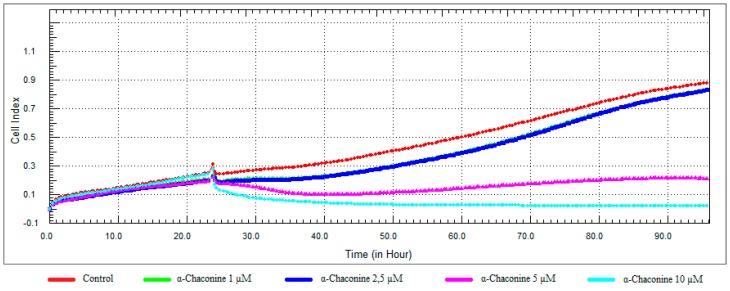
Dose and time-dependent cytotoxic effect and alteration of CI of α-chaconine determined by xCELLigence system: Effect of α-chaconine on proliferation of RL95-2 cell. Cells were treated with various concentrations of α-chaconine for 72 h. The CI was calculated from four repeated measurements.

**Figure 8:**
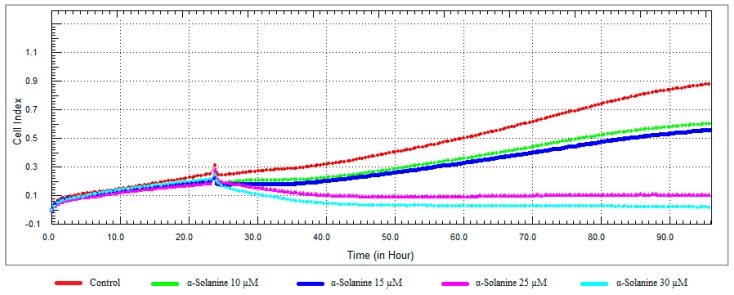
Dose and time-dependent cytotoxic effect and alteration of CI of α-solanine determined by xCELLigence system: Effect of α-solanine on proliferation of RL95-2 cell. Cells were treated with various concentrations of α-solanine for 72 h. The CI was calculated from four repeated measurements.

**Figure 9:**
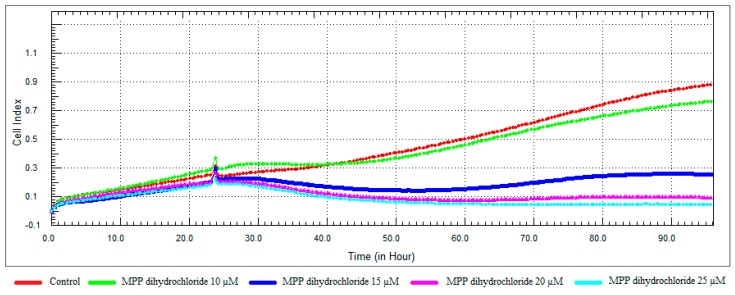
Dose and time-dependent cytotoxic effect and alteration of CI of MPP dihydrochloride determined by xCELLigence system: Effect of MPP dihydrochloride on proliferation of RL95-2 cell. Cells were treated with various concentrations of MPP dihydrochloride for 72 h. The CI was calculated from four repeated measurements.

**Figure 10:**
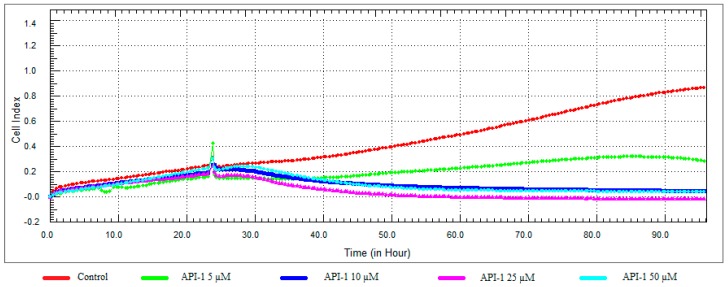
Dose and time-dependent cytotoxic effect and alteration of CI of API-1 determined by xCELLigence system: Effect of API-1 on proliferation of RL95-2 cell. Cells were treated with various concentrations of API-1 for 72 h. The CI was calculated from four repeated measurements.

To monitor and validate the reliability and accuracy of the SRB assay in evaluating the cytotoxic effects of the compounds, the cell viability was evaluated using the xCELLigence system in parallel with the studies above. As the cells interacted with these compounds, continuous CI alterations resulting from changes of cell number, morphology, and adhesion on the microelectrodes were measured by the RTCA SP instrument for approximately 72 h. A correlation was noted between results obtained from the impedance-based detection and those from SRB assay. As shown in Figure 3, Figure 4, Figure 5 and Figure 6b, the concentration-response curves of the given time points marked with dashed lines in Figure 7, Figure 8, Figure 9 and Figure 10 corresponded well with at the same dose of the xCELLigence system. The concentration-response curves and the viability of treated cells at the indicated time points are exhibited in Figure 7, Figure 8, Figure 9 and Figure 10. Control groups without compound treatment indicated the normal cell growth in electronic microwells. As shown in Figure 7, the CI values of the treated cells decreased from 5 µM in a concentration-dependent manner after an initial rise, while the CI values of the control kept rising until the maximal value at about 72 h after the treatment. In order to compare the cytotoxic effects of the four compounds, their 50% inhibitory concentrations (IC_50_) were calculated after 24-h exposure. The IC_50_ values of α-chaconine, α-solanine, API-1 and MPP dihydrochloride were 4.72, 26.27, 56.67 and 20.01 μM, respectively (Table 1). 

**Figure 11:**
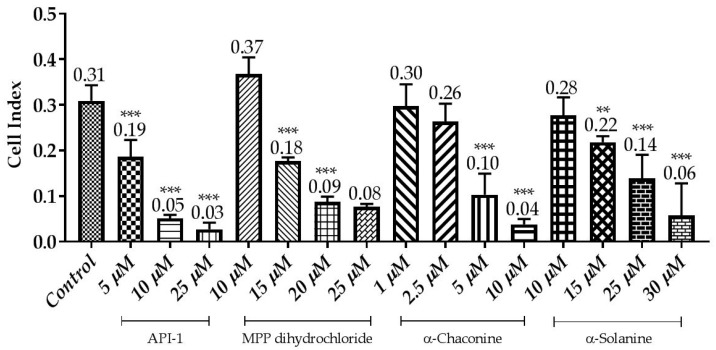
Correlation between cell index and the compounds effects using the xCELLigence system. Cells were treated with various concentrations of the compounds for 24 h. The CI was calculated from four repeated data and presented as mean ± SD. ** *p* < 0.01 and *** *p* < 0.001 compared with the untreated control.

α-Chaconine has an antiproliferative effect at concentrations of 1 and 2.5 μM; it was observed that cell viability decreased at concentrations of 5 and 10 μM in a dose-dependent manner (Figure 7 and Figure 11). RTCA viability assay results showed similar profiles at 10 μM concentrations (Figure 5b), while 2.5 μM α-chaconine reduced viability by at least 70%. A decision was made to work with this concentration since the CI value was 50% change at 5 μM and was close to IC_50_ (4.71 µM).

It was observed that 10 and 15 μM concentrations of α-solanine had an antiproliferative effect and decreased cell viability at 25 and 30 μM concentrations (Figure 8 and Figure 11). RTCA exhibited similar profiles to the control at concentrations of 10 and 15 μM according to the viability test results. α-Solanine did not provide a 50% CI change at 25 μM concentration and was close to a value of 26.27, which was calculated as the IC_50_ value, therefore 30 μM concentration has been selected for further studies.

MPP dihydrochloride showed antiproliferative activity at a concentration of 10 μM, while cell viability decreased at concentrations of 15, 20 and 25 μM in a dose-dependent manner (Figure 9 and Figure 11). According to RTCA and SRB results, IC_50_ value was calculated 20 μM for MPP dihydrochloride and this concentration was selected for further studies.

While 5 μM concentration of API-1 showed antiproliferative activity, cell viability was reduced at 10, 25 and 50 μM concentrations and the CI value at 25 μM concentration decreased to below 0.05 (Figure 10 and Figure 11).

SRB and real-time xCELLigence measurements show that RL95-2 cells are more sensitive to α-chaconine then the other tested compounds (Table 1).

### 3.4. α-Chaconine, α-Solanine Inhibits Phosphorylation of Akt (Ser473) and ERα

Since Akt is crucial for cancer progression, being a critical mediator and required for metastasis, the effect of the glycoalkaloids were investigated on the activation of Akt.

**Figure 12:**
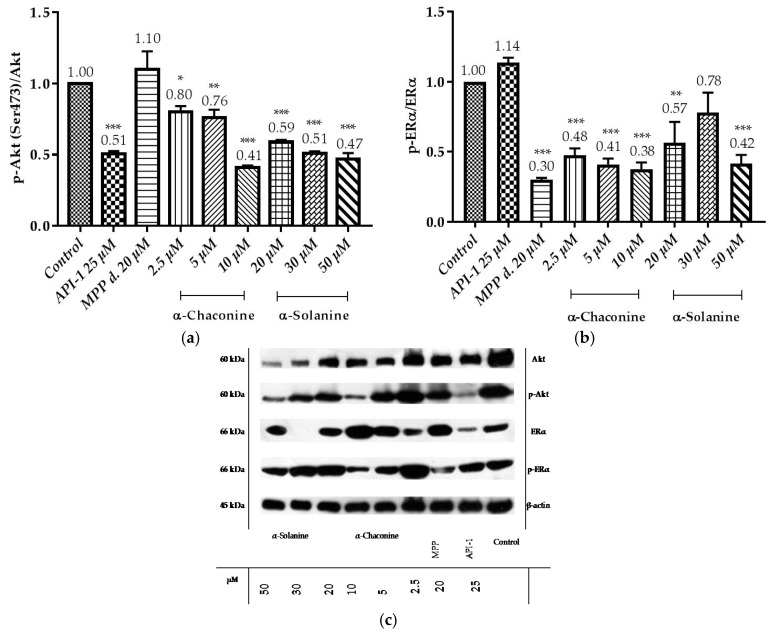
Western blot analysis of the expression of p-Akt, Akt, p-ERα and ERα proteins in the RL95-2 cell line: (**a**) In the bar graph the data represent the relative density of the bands p-Akt (Ser473)/Akt; (**b**) In the bar graph the data represent the relative density of the bands p-ERα/ERα; (**c**) RL95-2 cells were treated with the compounds. The relative intensity is presented as mean ± SD. three independent experiments. * *p* < 0.1, ** *p* < 0.01 and *** *p* < 0.001 compared with the untreated control.

Data demonstrated that API-1 reduced the phosphorylation of Akt (Figure 12a,c), while it did not alter the phosphorylation of ERα (Figure 12b,c). MPP dihydrochloride reduced the phosphorylation of ERα, while it did not alter the phosphorylation of Akt. The quantitative results showed that α-chaconine significantly suppressed the phosphorylation of Akt and ERα in a dose-dependent manner (Figure 12a–c). α-Solanine significantly suppressed the phosphorylation of Akt in a dose-depend manner except for 30 μM (Figure 12b,c). 

### 3.5. Changes in the Expression of Akt and ERα Genes

**Figure 13:**
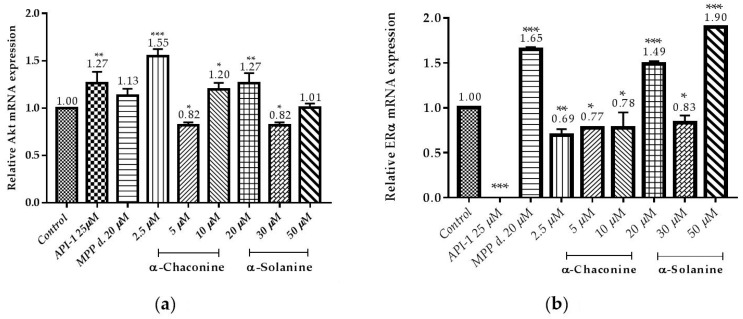
Effects of α-chaconine and α-solanine on suppressing the genes expression of: (**a**) Akt; (**b**) ERα in the RL95-2 cell line. The mRNA expressions were calculated from three repeated data and presented as mean ± SD. * *p* < 0.1, ** *p* < 0.01 *** *p* < 0.001 compared with the untreated control.

Expression of genes associated with cancer progression was demonstrated by RT-qPCR (Figure 13a,b). It is demonstrated that α-chaconine and α-solanine suppressed the expression of Akt mRNA at concentration 5 µM and 30 µM, respectively as seen in Figure 13a. The results showed that API-1, α-chaconine and 30 µM α-solanine markedly suppressed the mRNA expression of ERα (Figure 13a,b). 

## 4. Discussion

The most basic steroidal trisaccharide glycoalkaloids are α-chaconine and α-solanine that are found in potato plants [16,20]. Solanidine, the aglycone of α-chaconine and α-solanine, shows structural similarities with diosgenin, the precursor of steroidal hormones. Solanidine is a nitrogen-containing equivalent of diosgenin (steroidal saponin) [42]. Although the potential toxicity of α-chaconine and α-solanine is well known, studies showing that they have beneficial effects such as anticholinesterase [43], anti-inflammatory [8], antibacterial [44], antiviral [10], antifungal [45], antimalarial [46] and anticancer [2] depending on dosage and use conditions are included in the literature. 

α-Chaconine and α-solanine can cause toxicity in mouse and normal human liver cells in physiological functions [18,47]. Although the steroidal glycoalkaloids are toxic to normal cell membranes and lead to cell disruption [48], the compounds are thought to be possesses with the potential for therapeutic treatment against cancer cells. α-Chaconine exhibits anti-carcinogenic potential, including the ability to inhibit cell growth of various cancer cell lines in the human colon [49], lung [50,51], prostate [17], liver [18,52], cervical [18] cancer cells. Similarly, α-solanine exhibits anti-carcinogenic potentials, such as inhibiting cell growth of various cancer cell lines in human melanoma [20], prostate [2,52], pancreatic [21] and mice breast [22], lung [51] cancer cells. However, no information was available in the literature regarding whether it is effective in combatting endometrial cancer, and the effects on endometrial cancer were investigated for the first time in this study.

In endometrial cancer, inhibition of both extrinsic (Fas proteins) and intrinsic (Bcl-2 protein family) apoptotic pathways, alterations in PI3K/Akt activity and p53 mutation are known resistance mechanisms. Progressive and recurrent endometrial carcinomas continue to be a compelling tumor group with a high rate of multi-factor chemotherapy resistance. Chemotherapy resistance resulting from overexpression of drug flow efficacious proteins and mutations in β-tubulin isoforms in both primary and recurrent disease represent treatment difficulties. For this reason, the need for a less sensitive new agent for known resistance pathways arises [53]. The development of resistance to these drugs and the serious toxic effects of high doses lead researchers to search for different drug molecules. 

When the effects of α-chaconine and α-solanine on cell viability is examined, most of the studies have been performed on various cell culture lines and confirm our findings [16,20,50,54]. The IC_50_ values of α-solanine in HepG2, human gastric carcinoma (SGC-7901) and human large intestine cancer (LS-174) cells were 14.47, >50 and >50 μg/mL, respectively, and HepG2 cells were more sensitive to α-solanine [55]. The IC_50_ value of α-solanine was found to be 34 μM in mouse mammary carcinoma cells (4T1) and 30 μM in human hepatocellular carcinoma cells (HuH-7) [22]. These findings agree with the IC_50_ value of our study of α-solanine. In our study, glycoalkaloids were not combined. However, it is shown that α-chaconine has a more acute toxicity in RL95-2 than α-solanine. According to our findings, α-chaconine increased cell viability at concentrations of 1 μM and lower at 10 μM and lower concentrations of α-solanine, similar to steroidal hormones [56] in the structure of solanidine, the aglycone of these glycoalkaloids, it was evaluated that they could be responsible for this effect.

Since α-chaconine and α-solanine have decreased both cell proliferation and apoptosis-inducing effects in different cancer types as mentioned above, we decided to investigate the effects of these compounds with API -1 and MPP in regulation of Akt and ERα signaling in estrogen-positive RL95-2 cells.

In this study, SRB cytotoxicity assay was chosen to give better linearity and signal-to-noise ratio than sensitive, simple, and formazan-based experiments when measuring the effect of α-chaconine and α-solanine on viability in RL95-2 cells [57]. 

Supporting the previous data with α-solanidine with MCF-7 cells, α-solanine significantly increased the viability relative to control group up to a concentration of 10 μM [56].

We showed that α-chaconine and α-solanine reveal similar effects at different concentrations and these differences might be their glycone group, although the aglycones are the same. It is also possible to discover more effective molecules by synthesizing chemical derivatives. The inhibition is related to a concentration—but not time—dependent manner. Both compounds might have an even stronger effect than API-1 and MPP dihydrochloride, although there was no significant difference between their IC_50_ values in this study. Their cytotoxic efficacy on RL95-2 cells was α-chaconine > MPP dihydrochloride > α-solanine > API-1. Thus, more studies should be conducted to investigate the activity of these compounds especially these steroidal glycoalkaloids. In this in vitro study, the activity of these glycoalkaloids and the potential structure–activity relationship was shown, and further in vivo investigation about the cellular mechanisms should be conducted in the future. As shown in Figure 5a and Figure 6a, all the two steroidal glycoalkaloids have same aglycone but not glycone side chain. The cytotoxic effects of α-chaconine which contains chacotriose was significantly stronger than α-solanine with solatriose groups. Thus, the glycone groups besides aglycone ring might be an influencing factor for the cytotoxicity effects of potatoes steroidal glycoalkaloids on RL95-2 cells. There is another study in the literature where this effect is seen in different cell lines supporting our results [19].

When the effects of both glycoalkaloids on cell viability is examined, some of the studies have been performed on various cell culture lines and confirm our findings. From the results obtained, it can be concluded that α-chaconine can decrease phosphorylation of ERα as the same significance in positive control MPP dihydrochloride on RL95-2 cells whereas the specific Akt inhibitor API-1 did not show any effect on ERα phosphorylation. It has been shown for the first time that it may be related to the steroidal aglycone solanidine structure that they might have on the estrogen-dependent pathways on endometrium cancer cell line. This study showed for the first time the effect of α-chaconine on cell proliferation, ERα activity and expression in RL95-2 cells.

It has been shown that α-chaconine inhibits bovine aortic endothelial cell proliferation, migration and invasion. These effects have been shown to be mediated by JNK and PI3K/Akt signaling pathways and NF-κB activation by antiangiogenic activity [20]. α-Chaconine also showed anti-metastatic activity in human lung cancer cells A549. This effect has been shown to decrease MMP-2 and MMP-9 activities [50]. In another study, α-chaconine inhibited prostate cancer cells proliferation (LNCaP and PC-3) by increasing p27 levels and downregulating Cyclin D1, and apoptosis in these cells was associated with caspase-dependent and independent pathways. In addition, it has been shown that caspase-dependent apoptosis is induced via JNK activation in that study [17]. Beforehand α-solanine has been shown to inhibit metastasis, migration and invasion in human melanoma cells (A2058) by inhibiting JNK, PI3K, Akt phosphorylation and NF-κB activation and by decreasing MMP-2/9 activity/expression [20]. In another study, α-solanine down-regulated oncogenic microRNA-21 expression by decreasing MMP-2/9 expression via the ERK and PI3K/Akt signal pathway suppressed by human prostate cancer cell (PC-3) invasion and upregulated of tumor suppressor microRNA-138 [2]. α-Solanine has been shown to increase expression of radio-sensitivity and chemo-sensitivity by decreasing miR138 and focal adhesion kinase (FAK) expression in lung cancer cells (A549 and H1299) [58]. It has been reported in the literature that α-solanine acts as an antitumor agent in inhibition of Wnt/β-catenin, Akt/mTOR, Stat3 and NF-κB pathways in non-toxic concentrations in healthy cells and in pancreatic cancer cells (PANC-1) [21]. Another study has shown that α-solanine induces apoptosis by reducing the ratio of Bcl-2/Bax resulting in intracellular [Ca^2+^]i, which can cause and alteration in the enzymatic activity of the caspase family in human hepatocarcinoma cells (HepG_2_) [21]. In a study of the effects of α-solanine on the mitochondrial membrane potential and intracellular [Ca^2+^]i in HepG2 cells, α-solanine resulted in lowering the membrane potential and reducing the Ca^2+^ concentration in the organs by facilitating the opening of the permeability transition (PT) channels in the mitochondria. This has been shown to increase the Ca^2+^ concentration in the cell and thus trigger the apoptosis mechanism [59]. Therefore, α-chaconine and α-solanine are thought to play an active role in the control of signal transduction pathways and signaling proteins and apoptosis-dependent or independent cell proliferative functions. However, so far, the mechanism of these steroidal glycoalkaloids actions and effects in endometrium cancer has not yet been elucidated. In this study it is found that α-chaconine may have led to an increase in cell viability related to an increase in mRNA overexpression of Akt compared with the control, but the level of growth factors in the environment is increased at the 2.5 μM concentration and should be investigated with different parameters in detail. The reason for preference for the investigation of Akt expressions of RL95-2 cells is the presence of active Akt in mutated PTEN human endometrial cancer cells [60]. In our study, it was found that API-1 25 μM strongly reduced p-Akt (Ser473) levels, resulting in RL95-2 cells sensitive to API-1. However, the relative p-Akt (Ser473) protein level results at a concentration of 10 μM α-solanine at 20, 30 and 50 μM α-chaconine were shown to be at the same level of significance as the 25 μM reduction of API-1. Thus, at these applied concentrations, α-chaconine and α-solanine were found to be as potent as API-1, the pure Akt inhibitor, inhibition of Akt phosphorylation. In our study, it was observed that 10 μM α-chaconine and 20, 30 and 50 μM α-solanine were effective in decreasing p-Akt (Ser473)/Akt ratio compared to control 25 μM API-1. At these concentrations, Akt was shown to act by reducing phosphorylation like API-1. This effect of α-chaconine and α-solanine was shown to be related to the dose and these findings were also confirmed in the RL95-2 cell of the α-chaconine A549 [50] and α-solanine [21] in the PANC-1 cells. 

When the p-ERα/ERα ratios were evaluated, it was observed that α-chaconine (2.5, 5 and 10 μM), α-solanine (50 μM) and the specific ERα inhibitor was as effective as MPP dihydrochloride (20 μM) to reduce the ratio of p-ERα/ERα compared to the control group. At these concentrations, ERα, such as MPP dihydrochloride, was shown to be effective by reducing the phosphorylation. It has been shown for the first time that it may be effective in estrogen-dependent pathways and may be related to the steroidal aglycone solanidine structure. α-Chaconine showed this effect in a dose-dependent manner.

E2 stimulates the growth of many cancer cells. The mechanisms underpinning this effect include the blockade of MAPK/ERK and PI3K/Akt pathways that inhibit E2-induced DNA synthesis [34]. Estrogen-mediated signaling pathways are classified as genomic and non-genomic, depending on whether ER-dependence is transcriptionally regulated or not [61,62]. Taking into account the fact that RL95-2 cells are ER positive, we have investigated the effect of the compounds on the p-ERα/ERα expressions in these cells. In our study, p-ERα levels were increased α-chaconine (2.5, 5 μM) and α-solanine (20 μM) compared to the control group, and the increase in the concentration of α-chaconine 2.5 μM was found to be significant. This result is also consistent with the results of the RTCA and concludes that α-chaconine did not show cytotoxic effects up to 2.5 μM but may increase ERα phosphorylation through an estrogen receptor-dependent signaling pathway. However, contrary to protein expression, this decrease in mRNA level relative to control may be attributed to non-transcriptional activity of α-chaconine via non-genomic pathway using ERα interaction proteins and secondary messengers. This effect can also be caused by the estrogen-like effect of these steroidal compounds. The fact that RL95-2 cells are also ER positive confirms these results. The mRNA levels of Akt and ERα were decreased with α-chaconine and α-solanine at IC_50_ concentrations 5 and 30 μM respectively however these alterations were not concentration-dependent.

There are also limited data regarding the combination for these glycoalkaloids revealing that they have synergic toxic effects [63,64]. However, in our study the effects of the combination of these compounds has not been studied. 

Furthermore, bioavailability of these compounds is not well-defined and there are a limited number of papers regarding the bioavailability of these glycoalkaloids [63,65,66]. Because of the steroidal structure and lipid solubility of the compounds they might probably go through pre-systemic elimination or enterohepatic cycle therefore the bioavailability of the compounds should be clarified with further in vivo studies.

Our study shows that α-chaconine and α-solanine have cytotoxic effects in RL95-2 cells, specifically affect cellular signaling pathways, and decrease phosphorylation of Akt and ERα. It is clear that there is a need for other scientific studies to be able to fully elucidate which mechanisms might be related to other effects. In addition, the scope of this study should be supported by expanded in vitro and in vivo studies. In this respect, α-chaconine and α-solanine are thought to be potential candidates for endometrial cancer therapy research. 

## 5. Conclusions

In conclusion, we attributed the decrease in expression and activity of Akt and ERα by α-chaconine and α-solanine, and such suppressive effect might contribute to the inactivation of the PI3K/Akt and ERα signaling pathways in human endometrium cancer cells by both these steroidal glycoalkaloids. We demonstrated with a RL95-2 endometrial carcinoma cell line that α-chaconine and α-solanine alone seems to be as effective as both API-1 and MPP dihydrochloride. In ER positive cancers, active ER signaling is a pharmacological target so that ERα is a clinically important target for endometrial cancer. It is, therefore, necessary to carry out further research to explore novel candidates capable of both anti-estrogenic and cytotoxic potential. This should lead a strong insight into endometrial cancer therapy. In addition to this, steroidal glycoalkaloids which have anticancer potential to the estrogens, may also give further insight into endometrial cancer therapy. These findings reveal a new therapeutic potential for these glycoalkaloids on endometrial cancer therapy.

One of the main problems that should be taken into account while using such steroidal glycoalkaloids is the possible hormonal effects/interferences of these compounds on the physiological hormonal system. However, taking into account that conventional hormonal therapies are still being used for ERα positive endometrium cancer treatment, physicians should consider the benefit/risk ratio while treating these types of cancer, as this glycoalkaloids might be used to reduce the interference of these conventional hormonal therapies by lowering the doses with their synergistic effect which should be further investigated by in vivo studies.

On the other hand, it is possible to investigate whether pre- and combined estradiol administration to these glycoalkaloids leads to a change in Akt and ER expressions, and how it affects other endometrial cancer cell lines, Ishikawa and HEC-1A. When PI3K/Akt is thought to be one of the major signaling pathways in endometrial cancer, it is possible to investigate other signaling proteins associated with this pathway. It is also important to investigate whether the effects of these glycoalkaloids on cell migration are related to steroidal structural similarities.

## Figures and Tables

**Table 1:** IC_50_ values of RL95-2 cells for 24 h ^1^.

Compound	24 h
α-Chaconine	4.72 µM
α-Solanine	26.27 µM
API-1	56.67 µM
MPP dihydrochloride	20.01 µM

^1^ The IC_50_ of the four compounds were obtained based on the dose–response curves of CI during 24 h exposure in and calculated from repeated experiments (*n* = 4) with the real-time xCELLigence system.
